# Disseminated Human Parvovirus B19 Infection Induced Multiple Organ Dysfunction Syndrome in an Adult Patient With Alcoholic Hepatitis Complicated by Hemolytic Anemia: A Case Report and Literature Review

**DOI:** 10.3389/fimmu.2021.742990

**Published:** 2021-12-14

**Authors:** Jinmei Luo, Jingcong Zhang, Wenxing Lai, Shaofang Wang, Laizhi Zhou, Yunfeng Shi, Junhui Ba, Jiajia Hu, Yanhong Wang, Laisheng Li, Ben-Quan Wu

**Affiliations:** ^1^ Department of Internal Medicine, Medical Intensive Care Unit and Division of Respiratory Diseases, Third Affiliated Hospital of Sun Yat-sen University, Guangzhou, China; ^2^ Division of Hematology, The Third Affiliated Hospital of Sun Yat-sen University, Guangzhou, China; ^3^ Department of Laboratory Medicine, The First Affiliated Hospital of Sun Yat-sen University, Guangzhou, China

**Keywords:** human parvovirus B19, erythema infectiosum, acute aplastic crisis, hemophagocytic syndrome, multiple organ dysfunction syndrome, alcoholic hepatitis, hemolytic anemia

## Abstract

**Background:**

*Human parvovirus B19* (*B19*) can cause acute hepatitis and is attributed to the high mortality of alcoholic hepatitis (AH)*. B19* infection is generally self-healing in previously healthy people, but it can cause fatal effects in some high-risk groups and increase its virulence and infectivity. Disseminated *B19* infection-induced multiple organ dysfunction syndrome (MODS) in patients with AH has not been reported yet. Here, we described *B19* viremia in an adult patient with AH accompanied by hemolytic anemia (HA), leading to disseminated infection and secondary MODS, as well as self-limiting *B19* infections in seven nurses caring for him. Meanwhile, we reviewed the literature on AH and *B19* infection.

**Case Presentation:**

A 43-year-old male patient with AH accompanied by HA was transferred to the Third Affiliated Hospital of Sun Yat-sen University, Guangzhou, China, on March 31, 2021. After supportive treatment, his transaminase and bilirubin levels were reduced, but his anemia worsened. He received a red blood cell (RBC) infusion on April 9 for hemoglobin (Hb) lower than 6 g/dl. On April 13, he suddenly had a high fever. Under empirical anti-infection, his high fever dropped and maintained at a low fever level; however, his anemia worsened. On April 25, he was transferred to the medical intensive care unit (MICU) due to severe pneumonia, acute respiratory distress syndrome (ARDS), acute aplastic crisis (AAC), and hemophagocytic syndrome (HPS), which were subsequently confirmed to be related to *B19* infection. After methylprednisolone, intravenous immunoglobulin (IVIG), empirical anti-infection, and supportive treatment, the lung infection improved, but hematopoietic and liver abnormalities aggravated, and systemic *B19* infection occurred. Finally, the patient developed a refractory arrhythmia, heart failure, and shock and was referred to a local hospital by his family on May 8, 2021. Unfortunately, he died the next day. Fourteen days after he was transferred to MICU, seven nurses caring for him in his first two days in the MICU developed self-limiting erythema infectiosum (EI).

**Conclusions:**

*B19* infection is self-limiting in healthy people, with low virulence and infectivity; however, in AH patients with HA, it can lead to fatal consequences and high contagion.

## Introduction

AH usually occurs after decades of heavy drinking (average intake, approximately 100 g per day) (1). The cardinal sign of AH is the rapid onset of jaundice. Other common signs and symptoms of AH include fever, ascites, and loss of proximal muscle strength. Typically, the liver is enlarged and tender (2). Approximately 40% of patients with severe AH die within 6 months after the onset of the clinical syndrome (1). Hypersplenism, in addition to portal hypertension and splenomegaly, can cause hemolytic anemia (HA), and rapidly progressive HA is a life-threatening form of anemia in patients with severe liver disease (3). *B19* infection has been well reported to be associated with acute hepatitis and fulminant liver failure and may underlie secondary HA, which is an important reason for the increased short-term risk of death from AH (1, 3–5).

*B19* is a small, non-enveloped, single-stranded DNA virus of the *Erythroparvovirus* genus within the *Parvoviridae* family (6, 7). Its genome contains approximately 5,600 nucleotides, which encode two minor capsid proteins (VP1 and VP2) and a single non-structural protein (NS1) essential for viral replication (8). VP1 has an additional 227 aa at the N terminus, known as VP1u (9). The virus first binds to neutral glycosphingolipoglobulin (GB4, also known as blood group P antigen), leading to the externalization of VP1u, and then interacts with other receptors to internalize the virus (10, 11). A distinctive feature of *B19* is that it has a significant tropism to primary human erythrocyte progenitor cells (EPCs) in the bone marrow after respiratory transmission (12, 13). In addition to being mainly transmitted through the respiratory tract, the virus can also be transmitted through vertical transmission, blood transfusion, stem cell or solid organ transplantation, etc. (7).

The prevalence of IgG anti-*B19* antibodies (Abs) in the general population ranges from 2% to 21% in children (1–5 years), 30%–40% in adolescents (15 years), and 40%–60% in young adults (20–40 years) and reaches a maximum in the elderly, with a prevalence of over 90% (8, 14). The clinical manifestations of *B19* infection in immunocompetent individuals may be different, generally without any clinical symptoms or resulting in a self-limiting flu-like disease, erythema infectiosum in children, erythema infectiosum complicated by arthropathy or acute aplastic crisis (AAC) in adults, and hydrops in pregnant women (8, 15, 16). In patients with hematologic diseases, *B19* infection can cause autoimmune HA, neutropenia, thrombocytopenia, bicytopenia, pancytopenia, hemophagocytic syndrome (HPS), acute pure red cell aplasia (PRCA), and/or aplastic crisis (AC) (16–20). In addition, *B19* infection has also been reported to cause severe complications, such as liver diseases (elevated transaminase, acute hepatitis, fulminant liver failure, and fibrosing cholestatic hepatitis), respiratory diseases [interstitial lung disease, severe pneumonia, and acute respiratory distress syndrome (ARDS)], kidney diseases (acute glomerulonephritis and nephropathy syndrome), heart diseases [myocarditis or heart failure (HF)], and nervous system involvement, especially in patients with abnormal hematological parameters or immune status (4, 5, 16–24). However, we could not find any report of almost all relevant manifestations of *B19* infection, except in one patient, and here is such a patient.

## Case Presentation

A 43-year-old Chinese male veteran with a history of drinking for 20 years (80–150 g per day) presented to our hospital. He complained of fatigue, bad appetite, dark urine, and yellowish sclerae for 1 month. He neither had fever nor any other disease history, including hereditary or familial clustered infectious diseases.

**Figure 1:**
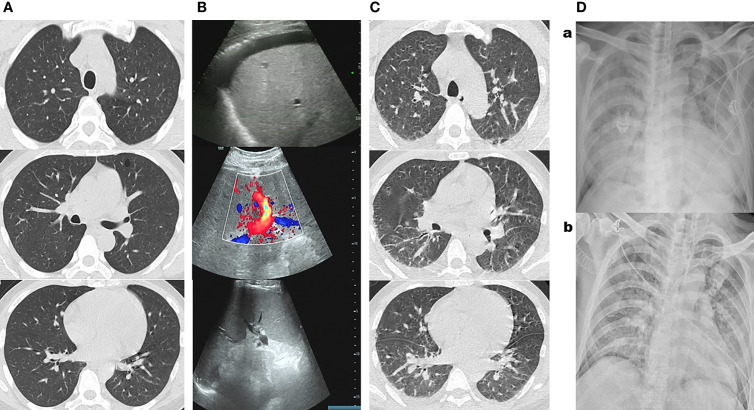
**(A)** Chest CT examination at admission showed no obvious abnormalities. **(B)** Chest CT showed a few scattered vague exudates and small nodules at midnight on the 26th day of admission before the patient was transferred to the medical intensive care unit (MICU). **(Ca)** Bedside chest radiograph showed consolidations, bilateral involvement, peripheral distribution, lower zone dominance, and reduction in lung volume on the third day in MICU. **(Cb)** Bedside chest radiograph showed lung lesions were significantly improved on the eighth day in MICU. **(D)** Abdominal color Doppler ultrasound reported fatty liver, alcoholic liver disease, dilated portal vein, and hepatosplenomegaly and ascites at admission.

**Figure 2:**
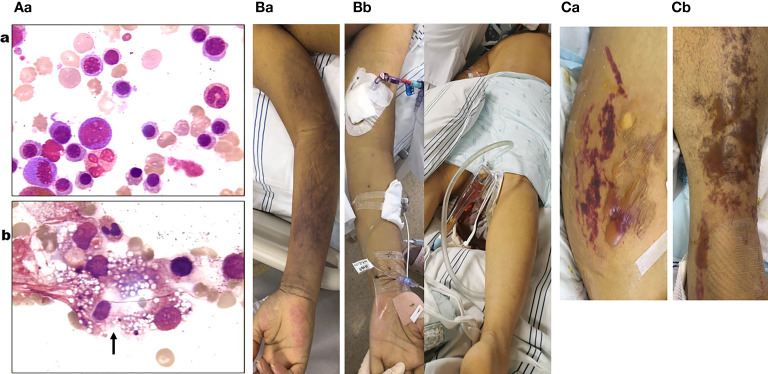
**(Aa)** Blue–purple ecchymosis on the proximal right upper limb of the patient when being transferred to medical intensive care unit (MICU). **(Ab)** Blue–purple ecchymosis did not appear on his trunk, lower limbs, and upper left limb at that time. **(Ba)** Strained blisters filled with serous fluids on predominantly erythematous skin on his torso (**Ba**; buttocks as a representative) and limbs [**(Bb)**; left limb as a representative] on the tenth day in MICU. **(Ca)** Bone marrow examination on the fourth day of admission (smear; ×400) revealed active bone marrow hyperplasia; a significant increase of red blood cells (RBCs) (52.25%) of which the middle and late stages of RBC account for a large proportion; no obvious abnormalities of granulocytes; lymph proportion was reduced with the morphology roughly normal; platelets were significantly reduced and distributed in single or clusters, with no clue to acute aplastic crisis or hemophagocytic syndrome. **(Cb)** Bone marrow examination on the second day in MICU (smear; ×400) revealed a marked decrease in the density of erythroblasts, inhibition of megakaryocyte maturation, and easily seen phagocytes containing vacuoles, platelets, impurities, and other components (black arrow).

**Figure 3:**
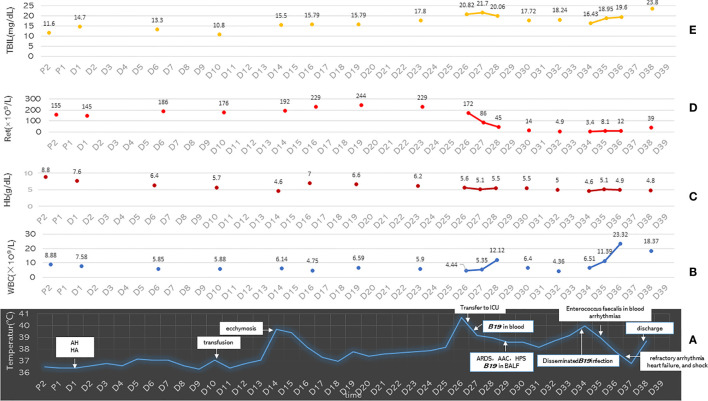
**(A)** The distribution of the main clinical events and body temperature of the patient on the time axis. **(B–E)** The Ret count, Hb, WBC, and TBIL values of the patient on the time axis. P1, day 1 pre-hospital; P2, day 2 pre-hospital; D1 to D39, day 1 to day 39 of hospital admission; AH, alcoholic hepatitis; HA, hemolytic anemia; ARDS, acute respiratory distress syndrome; HPS, hemophagocytic syndrome; AAC, acute aplastic crisis; Ret, reticulocytes; Hb, hemoglobin; WBC, white blood cell; TBIL, total bilirubin.

Physical examination at admission showed the following: height 170 cm, weight 86 kg, temperature 36.5°C, blood pressure (BP) 104/68 mmHg, heart rate (HR) 80 beats/min, oxyhemoglobin saturation by pulse oximetry (SpO_2_) under room air 98%, clear consciousness, dull face, palpebral conjunctival pallor, moderate scleral icterus, normal breath sounds in both chest regions, normal heart sounds, mild liver and spleen enlargement, abdominal distension with shifting dullness, and liver palms (palmar erythema). Laboratory studies revealed progressive thrombocytopenia, anemia with elevated reticulocytes (Ret), and liver damage compared with those at local hospitals (**Supplementary Material Table 1**). Serum lactate dehydrogenase (LDH) was 432 mg/dl (normal reference range (NR), 71–231 mg/dl), and serum ferritin was 1,992 (NR, 10–260 ng/ml). The urine bilirubin was positive. Meanwhile, laboratory studies also showed that the serum levels of C-reactive protein (CRP), beta-1,3-glucan, and galactomannan were all within the reference range (NR), with slightly elevated levels of procalcitonin (PCT) and interleukin (IL)-6 (**Supplementary Material Table 1**). The serum levels of vitamins B12, folate, and iron (iron, ferritin, transferrin, and transferrin saturation) were all within NR. The direct antiglobulin test (DAT) was negative. The serum markers of liver cancer, lung cancer, gastrointestinal cancer, thalassemia and glucose 6-phosphate dehydrogenase (G6PD) gene test, thyroid function test, laboratory tests of autoimmune hepatitis, vasculitis, systemic lupus erythematosus and rheumatoid, type A to E hepatitis, HIV, syphilis, the DNA and IgM of Epstein–Barr virus (EBV), and the DNA and IgM of cytomegalovirus (CMV) were all negative. Chest CT examination showed no obvious abnormalities (**Figure 1A**). Abdominal color Doppler ultrasound revealed fatty liver, alcoholic liver disease (ALD), dilated portal vein, hepatosplenomegaly, and ascites (**Figure 1B**), which were consistent with the enhanced abdominal CT report performed 4 weeks earlier at the local hospital (without details). Cultures of ascites were negative, and ascite routine examination found 240 white blood cells (WBC) (**Supplementary Material Table 1**). Bone marrow smears suggested HA without AAC or HPS (**Figure 2Aa**). Based on the above, he was diagnosed with AH, acute-on-chronic liver failure (ACLF), chronic cholecystitis, and HA. Under empirical antibiotics (cefoperazone/sulbactam) and supportive drug treatments, his transaminase and bilirubin levels decreased; however, anemia, thrombocytopenia, and abnormal blood coagulation worsened (**Supplementary Material Table 1**). On the 10th day of admission, he received a transfusion of 2 units of washed RBC for Hb<60 g/L (**Figure 3A**).

On the 14th day of admission, the patient suddenly had a high fever of 39.6°C and blue–purple ecchymosis on his proximal right upper limb (without picture). From this day to the 25th day of admission, he received meropenem instead of cefoperazone/sulbactam sodium as an empirical antibacterial treatment, and his highest body temperature dropped from 39.6°C to 38.2°C after 2 days, and then his fever was maintained at a low level (**Figure 3A**). During this period, laboratory tests showed that routine blood tests of WBC was within NR (**Supplementary Material Table1** and **Figure 3B**), and serum levels of CRP, PCT, beta-1,3-glucan, and galactomannan did not change much compared with those of before, and the serum IL-6 level was slightly higher (**Supplementary Material Table 1**). The WBC count in ascites was lower, with a higher proportion of granulocytes. Blood tests for influenza A and B, CMV, EBV, respiratory syncytial virus, rhinovirus, mycoplasma, chlamydia, and 2019-nCoV were all negative. Cultures and mass spectrometry of urine, ascites, and blood were all negative. However, during this period, in the case of blood transfusion, Hb showed a significant reduction with increasing Ret (**Figures 3C, D**), Platelet (PLT) was still maintained at a significantly low level, and the bilirubin gradually increased, and prothrombin activity (PTA) and concentration of fibrinogen (FIB) gradually decreased (**Figure 3A** and **Supplementary Material Table 1**). Arteriovenous color Doppler ultrasound of both upper limbs showed no abnormalities.

**Figure 4:**
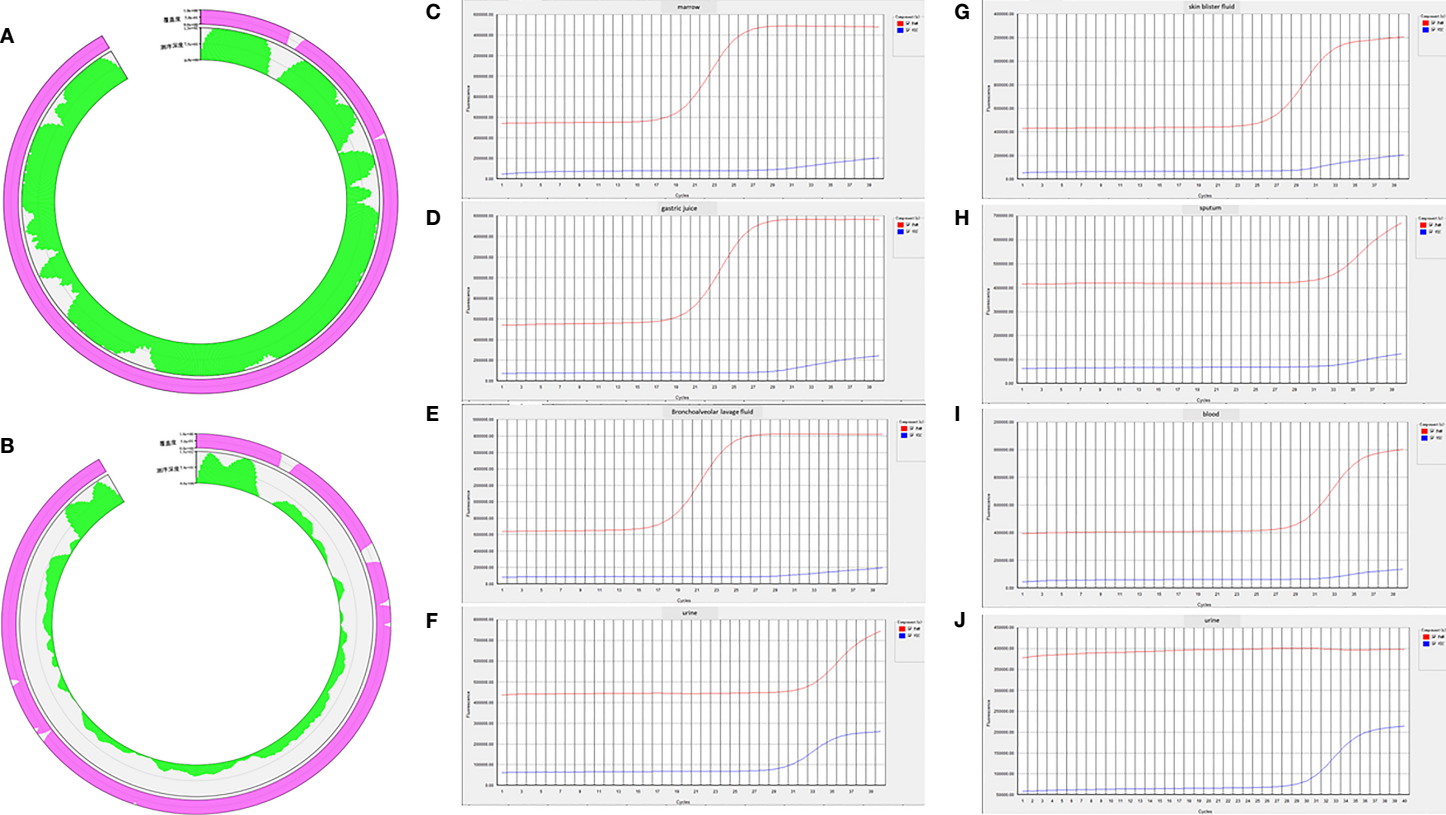
**(A)** Next-generation sequencing (NGS) results of blood collected at 8 h after the patient was transferred to the medical intensive care unit (MICU) reported extremely high DNA copies of B19 (1,605,726 copies/µg DNA, and the relative abundance was 99.99%). **(B)** NGS results of bronchoalveolar lavage fluid (BALF) collected on the third day in MICU reported high DNA copies of B19 (15,938 copies/µg DNA, and the relative abundance was 97.85%). **(C–F)** Real-time PCR confirmed positive results of B19 in bone marrow, gastric juice, BALF, and urine collected on the eighth day in MICU. **(G–J)** Real-time PCR confirmed positive results of B19 in skin blister fluid, sputum, and blood collected on the eighth day in MICU. **(J)** Real-time PCR showed that the previously positive result of B19 in urine became negative in the urine sample collected on the tenth day in MICU.

At midnight on the 26th day of admission, the patient’s temperature suddenly reached 40.7°C (**Figure 3A**), and the blue–purple ecchymosis on his proximal right upper limb enlarged (**Figure 2Ba**), however, Blue-purple ecchymosis did not appear on his trunk, lower limbs and upper left limb (including the catheterization sites of arteries and veins, **Figure 2Bb**). Simultaneously, he had shortness of breath, dyspnea, and cough without expectoration. His SpO_2_ decreased to 88%. Arterial blood gas (ABG) showed arterial oxygen partial pressure (PaO_2_) of 91.9 mmHg and arterial partial pressure of carbon dioxide (PaCO_2_) of 32.6 mmHg under low-flow nasal cannula therapy. Blood laboratory tests found that WBC, CRP, PCT, and galactomannan did not change much, and LDH and ferritin increased to 3,038 mg/dl and 1123 ng/ml, respectively. Serum IL-6 and beta-1,3-glucan were much higher than before, and the Ret% decreased from the original abnormally increased level to within NR (**Supplementary Material Table 1**). Chest CT showed a few scattered vague exudates and small nodules (**Figure 1C**). He was transferred to the medical intensive care unit (MICU). The Acute Physiology and Chronic Health Evaluation (APACHE-II) and Sequential Organ Failure Assessment (SOFA) scores were 14 and 16, respectively. For suspected viral or fungal infections, voriconazole was added as antifungal therapy, and a high-flow nasal cannula (HFNC) was given to relieve his shortness of breath and dyspnea. Blood transfusion-related infectious diseases including B19 infection was suspected by reviewing his medical history. A blood sample was collected for detection of B19 IgM and IgG and culture and next-generation sequencing (NGS) of pathogens on the second day (8 h after being transferred to the MICU). The next day, the NGS results reported extremely high DNA copies of B19 (1,605,726 copies/µg DNA, and the relative abundance was 99.99%) (**Figure 4A**).

In order to clarify the diagnosis of AAC (continuously decreasing RBC count and acute significant reduction of Ret count) and HPS (fever lasting 2 weeks, splenomegaly, increased ferritin, decreased FIB, anemia, and thrombocytopenia), on the second day in MICU, we performed a bone marrow smear and pathological biopsy on the patient. The results indicated the diagnosis of AAC with HPS (**Figure 2Ab**). Meanwhile, the blood smear showed a late stage and thereafter neutrophils, with the increasing neutrophil alkaline phosphatase (NAP) score, indicating the possibility of infection (without picture). Bone marrow biopsy immunohistochemistry and special staining showed that the islands of young RBCs were not easy to see, hemophagocytic cells containing RBCs were visible, and no tumor-related bone marrow manifestations were observed, indicating the possibility of AAC with HPS (without picture). Therefore, methylprednisolone (1 mg/kg) and IVIG (0.4 g/kg) were prescribed.

On the third day in the MICU, the patient experienced significantly worsening shortness of breath with profuse sweating, HR of 130 beats/min, respiratory rate of 35 breaths/min, SpO_2_ of 86%, BP of 120/76 mmHg, urine output of 230 ml/h, and many wet rales in the lungs. Bedside chest radiograph showed “consolidations, bilateral involvement, peripheral distribution, lower zone dominance and reduction in lung volume” (**Figure 1Da**). Bedside B-ultrasound showed a left ventricular ejection fraction (EF) of 65%. The myocardial enzyme spectrum was within NR, and ABG revealed PaO_2_ of 51 mmHg, arterial oxygen saturation (SaO_2_) of 83%, PaCO_2_ of 41 mmHg, alveolar-arterial oxygen gradient (A-aDO_2_) of 546, and lactic acid (Lac) of 8.7 mmol/L. The APACHE-II and SOFA scores increased to 32 and 28, respectively. Severe pneumonia and ARDS were newly diagnosed, and endotracheal intubation for invasive mechanical treatment was performed. Infection by B19 mixed with other pathogens in his lung was suspected. Smears, culture, and NGS of bronchoalveolar lavage fluid (BALF) were analyzed. His oxygenation did not improve under very high parameters of invasive mechanical ventilation (synchronized intermittent mandatory ventilation (SIMV): Vt 5 ml/kg, positive end-expiratory pressure (PEEP) 15 cmH_2_O, Ps 16 cmH_2_O, and FiO_2_ 90%), so prone ventilation was administered after adequate analgesia, sedation, and muscle relaxation. After 12 h of prone ventilation, his oxygenation improved, and ventilatory parameters decreased gradually. ABG showed that the Lac reduced to a normal level of 1.7 mmol/L, oxygenation improved significantly, and ventilatory parameters were decreased to a lower level (SIMV: Vt 5 ml/kg, PEEP 8 cmH_2_O, Ps 12 cm cmH_2_O, and FiO_2_ 60%).

On the fourth day in the MICU, NGS of BALF reported B19 (15,938 copies/µg DNA, and the relative abundance was 97.85%) (**Figure 4B**), Candida albicans (5 copies/µg DNA, and the relative abundance was 4.55%), Rothia mucilaginosa (86,379 copies/µg DNA, and the relative abundance was 82.66%), Streptococcus pneumoniae (2,727 copies/µg DNA, and the relative abundance was 11.42%), and Streptococcus mitis (1,657 copies/µg DNA, and the relative abundance was 99.99%). Cultures of blood and BALF reported negative results. During the next three days, the patient’s condition improved to some extent, ventilatory parameters were further decreased to a lower level (SIMV: Vt 5 ml/kg, PEEP 5 cmH_2_O, Ps 12 cmH_2_O, and FiO_2_ 44%), and ABG showed good ventilation and oxygenation of the lungs (PaO_2_ 141 mmHg, SaO_2_% 99.9%, PaCO_2_ 36 mmHg, A-aDO_2_ 113, and Lac 1.4 mmol/L). The previously increased transaminase, bilirubin, IL-6, and beta-1,3-glucan levels gradually decreased, but Hb, PLT, Ret count, and Ret% were still at very low levels (**Supplementary Material Table 1**). The APACHE-II and SOFA scores decreased to 23 and 26, respectively.

On the eighth day in the MICU, the patient again had a high fever (40°C) (**Figure 3A**), and scattered purple-red ecchymosis appeared on his limbs and trunk, which was initially considered to be related to poor liver and coagulation function. Routine blood tests found that the WBC count was within NR; however, Hb, PLT, Ret count, and Ret% were still at very low levels (**Supplementary Material Table 1**). FIB decreased to 0.88 mg/dl. Serum LDH increased to 3,486 mg/dl, and ferritin increased to 2,000 ng/ml. Bone marrow aspiration smears again showed AAC and HPS. Since the WBC count and serum levels of CRP, PCT, IL-6, galactomannan, and beta-1,3-glucan did not change much and lung lesions were significantly improved (**Figure 1Db**), bacterial and fungal infections were not considered at that moment; disseminated B19 infection besides previously confirmed infection in the blood and lung was suspected. To confirm this, bone marrow, gastric juice, BALF, and urine were collected and detected for B19 by real-time PCR. Positive results of all the specimens (**Figures 4C–F**) confirmed the correctness of inference.

On the ninth day in the MICU, routine blood tests showed significantly increased WBC (**Supplementary Material Table 1** and **Figure 3B**), and empirical vancomycin was given to cover cocci (blood culture later reported Enterococcus faecalis, which was sensitive to vancomycin). The patient’s body temperature decreased, and the blood culture result became negative (later reported); however, recurring obvious arrhythmias appeared without hypokalemia. Electrical cardioversion was implemented because of his poor response to cedilanid and amiodarone.

On the tenth day in the MICU, his torso and limbs developed strained blisters filled with serous fluid on predominantly erythematous skin (**Figures 2Ca** and **Cb**). B19-related bullae and bullous pemphigus were suspected. Bullous pemphigus-related anti-desmosomal core glycoprotein 1,3 antibodies (Dsg1 and Dsg3) and structural components of the hemidesmosome (BP180) were all negative, while real-time PCR confirmed B19 positivity in the skin blister fluid (**Figure 4G**), sputum (**Figure 4H**), and blood (**Figure 4I**), although the B19 DNA in the urine became negative (**Figure 4J**). On this day, his urine output decreased to 50–60 ml/h, his serum sodium increased to 178 mmol/L, and he still had persistent rapid atrial fibrillation and newly developed refractory HF responding poorly to anti-arrhythmic and anti-HF treatment (amiodarone, digitalis, recombinant human brain natriuretic peptide, and continuous renal replacement therapy). Acute myocarditis caused by B19 infection was considered. Although MRI and endomyocardial biopsy are important tools for the diagnosis of myocarditis, they were not under consideration, for the patient’s condition was too serious to hold up the life-threatening risk during the MRI examination and heart biopsy processes.

On the eleventh day in the MICU, his elevated body temperature and WBC decreased, and his Ret count and ratio rebounded; however, his bilirubin was higher, and his HF symptoms worsened, with the appearance of pink foamy serous sputum and shock needing vasoactive drugs (norepinephrine 0.2–0.4 μg/kg/min and dopamine 5–10 μg/kg/min) to maintain BP at 77–110/22–77 mmHg. His family gave up, and the patient was transferred to the local hospital, where he died the next day (**Supplementary Material Table1** and **Figure 3**).

## Discussion

From his onset to the 14th day of admission to our hospital, the patient mainly manifested liver injury and blood system damage (hyperbilirubinemia manifested as indirect bilirubin (IBIL) > direct bilirubin (DBIL), aspartate aminotransferase (AST) > alanine aminotransferase (ALT), coagulation dysfunction, thrombocytopenia, anemia with Ret significant increase in count) (**Supplementary Material Table 1**). Combined with the patient’s long-term heavy drinking and the later-confirmed B19 infection, alcoholic liver disease and *B19*-associated acute hepatitis should be differentially diagnosed.

Alcoholic liver disease covers a spectrum of disorders beginning with fatty liver and progressing at times to AH; more severe cases lead to liver failure and are diagnosed predominantly on clinical history, physical examination, and laboratory testing (1, 25). Although liver biopsy is usually required to ensure the diagnosis, severe cases are often contraindications to biopsy because of coagulation dysfunction and/or thrombocytopenia (1, 25). Prolonged alcohol abuse and the results of physical, laboratory, and auxiliary examinations in this patient fulfilled the diagnostic criteria of AH. However, for this patient, non-alcoholic steatohepatitis and B19-associated acute hepatitis should be distinguished from AH. The alcohol–non-alcoholic index (ANI) is a novel scoring system that is highly accurate in distinguishing alcoholic liver disease (ALD) from non-alcoholic fatty liver disease (NAFLD) and is most accurate when the model for end-stage liver disease (MELD) score is below 20 (1). However, MELD calculations may also be performed for the 90-days mortality rate of AH (2) ANI uses the body mass index (BMI), mean corpuscular volume (MCV), ratio of AST/ALT, and sex to determine whether alcohol is the etiology of liver disease. Usually, low BMI, high MCV, AST/ALT >1 (with AST rarely above 400 U/L), hyperbilirubinemia (total bilirubin [TBIL] >5 mg/dl), anemia, elevated international normalized ratio (INR), and neutrophilia in a patient with ascites, in addition to male sex and a history of heavy alcohol use, favor alcohol as the etiology (this is reflected as a positive ANI score; a negative score is good for NASH) (1, 3, 19). According to the ANI calculator available online (http://www.mayoclinic.org/girst/mayomodel10.html), the ANI score of this patient was 6.875, and the probability of ALD was 99.9%. The MELD score of this patient was 42 at admission, and 90-day mortality rate was 92%, which suggested that this patient was with severe AH.

Although *B19* infection can explain the patient’s illnesses from manifestations at disease onset to later-confirmed hepatosplenomegaly, liver damage, thrombocytopenia, and HA, it cannot be concluded that *B19* infected him at the beginning because of a lack of tests related to *B19*. However, we can be sure that *B19* caused the patient’s persistent high fever, sudden respiratory distress, and hypoxemia that required his transfer to the MICU and also his subsequent deterioration. ELISA confirmed antibodies against *B19* IgM and IgG in his serum specimen (specimen collected on his first day in the MICU), and NGS detected high B19 viral loads in the specimens of his blood (1.61 × 10^6^ copies/ml sera, **Figure 4A**, specimen collected on his second day in the MICU) and BALF (1.62 × 10^4^ copies/ml, **Figure 4B**, specimen collected on his third day in the MICU). Traditionally, a laboratory diagnosis of B19 infection relies on serologic and DNA tests (26). Patients with chronic HA are at high risk of developing acute erythroblastopenia following infection by the virus, and they usually become highly viremic (26). During B19 infection, viremia has its onset 5–6 days after infection and declines a few days later with the appearance of IgM antibodies against VP1 and VP2 proteins (27, 28). In general, IgM antibody appears about 10–14 days after infection and may be detected up to 2–3 months following infection (27, 28). In contrast, B19 IgG usually appears about 1–2 weeks after infection and then probably persists lifelong thereafter (29). As *B19* can be transmitted through blood transfusion (7), if the patient was infected with *B19* during the first RBC transfusion, his high fever and ecchymosis could be explained by the viremia produced 5–6 days after *B19* infection; and the production of IgM 10 days after the virus infection, thereby reducing the viral load, could also explain his subsequent improvement in body temperature. It is a pity that *B19* infection was not considered at the time, and related tests were neither carried out. The positive results of B19 IgM and IgG in the serum specimen collected on the first day of this patient in the MICU suggested that he was infected with B19 at least 1 week before being transferred to the MICU. This hinted that before being transferred to the MICU, his sudden high fever, aggravated erythroblastopenia, increased levels of IL-6, and increased levels of previously decreased bilirubin (**Supplementary Material Table 1**) could all be caused by B19 infection.

In addition to the higher *B19* viral loads and infectivity, patients with underlying diseases have a higher proportion of anemia, bicytopenia or pancytopenia, hepatosplenomegaly, and tachycardia (16). Patients with increased destruction of or a high demand for erythrocytes, such as HA or hereditary spherocytosis, may suffer from transient AC after infection with *B19* (16, 18). HA was confirmed before this patient had a fever by blood tests, blood smears, and bone marrow smears, and when he was transferred to the MICU because of his worsening condition, blood and bone marrow smears confirmed AC and HPS (**Figure 2Ab**), along with high copies of B19 in the blood and BALF of this patient. *B19* has been shown to increase the secretion of interferon gamma (IFN-γ) and tumor necrosis factor alpha (TNF-α) by recruiting striking CTLs21 that could trigger an autoimmune response, which can lead to autoimmune anemia (27, 28). The pathological mechanism of HPS in viral infection may be attributed to its ability to recruit CD8+ T cells that secrete IFN-γ and TNF-α, leading to hypersplenism and secondary HPS. In addition to a direct cytopathic effect, *B19* triggers the activation of macrophages, which play an important role in HPS (30–33). During times when patients are under immunosuppression or during infection with other pathogens, the viral load increases, causing extensive cell death of EPCs, leading to various inflammatory diseases (8). The wide distribution of *B19* receptors in human erythroid and non-erythroid tissues, such as hepatocytes, endothelial cells (vascular and intracardiac), placental trophoblastic cells, and some megakaryocytes cells, could explain the ability of *B19* to cause multiorgan diseases (15). The *B19* capsid binds its primary receptor, P antigen, and undergoes a conformational change, exposing VP1u, a unique (273 aa) N-terminus of the VP1 capsid protein. VP1u interacts with some unknown coreceptor for subsequent internalization. The VP1u region (without a capsid) is efficiently internalized by *B19*-permissive cells, which suggests that the primary interaction of *B19* with the P antigen is required only for externalization of VP1u. Mature RBCs also express P antigen and hence show primary attachment to *B19*, but the virus is not internalized. It is presumed that this primary interaction may be responsible for the systemic dissemination of the virus (12).

There is no specific treatment for *B19* infection, except IVIG treatment or blood transfusion (34). Under the treatments of antibacterial and antifungal drugs, IVIG, blood transfusion, and other supportive treatments, the high fever, liver damage, and lung lesions of the patient seemed to have improved; however, his HA and APS did not improve. Moreover, disseminated infection appeared, including that previously confirmed in the blood and lung and later confirmed in the bone marrow, digestive tract, urinary tract, and skin and the presumed liver damage and heart damage. High viral loads of *B19* enter the endothelial cells of intramyocardial arterioles and postcapillary venules, leading to increased expression of proinflammatory cytokines and apoptosis of endothelial cells. This sustained cardiac inflammation induced by continuously elevated cytokine levels is observed in approximately 60% of *B19*-positive DCM patients and results in endothelial dysfunction, impairment of the myocardial microcirculation, and, ultimately, myocyte necrosis (35). The clinical manifestations of acute myocarditis vary widely, from asymptomatic changes on electrocardiogram to fulminant heart failure, arrhythmias, and sudden cardiac death (36). MRI is emerging as an important tool for the diagnosis and follow-up of patients and guidance of endomyocardial biopsy (36). Combined with *B19*-related literature reports and the patient’s clinical manifestations and positive laboratory test results (including *B19* DNA detected in the patient’s blood, BALF, skin blister fluid, bone marrow, gastric juice, and urine), the patient should have been clearly diagnosed with disseminated B19 infection.

Patients with chronic HA infected by the virus usually have an increased risk of virus transmission (37). The virus can be transmitted primarily via the respiratory route. From the respiratory epithelium, the virus particles access the bloodstream by an unknown mechanism (7). Nosocomial transmission in hospital situations has been well documented, and patients with AC or persistent infection should be considered infectious (29). This patient with AC was diagnosed with B19 infection on his second day in MICU. This alerted the hospital that the patient may be a source of highly contagious B19; the nosocomial infection control team of the undergraduate center held an emergency meeting to study and discuss the structural characteristics of B19 and the infection’s epidemiology, pathogenesis, clinical manifestations, diagnosis, treatment, and prevention. At the same time, the physicians and care workers were also organized to learn about infectious disease-related guidelines (38–40). However, three days after his discharge, that is, two weeks after he was transferred to the ICU, the seven nurses taking care of him during his first two days in the MICU all developed a purpuric rash with itching, and 4 of them had joint swelling and pain. They were suspected of being infected with *B19*. Due to the formation and deposition of immune complexes in the skin after B19 infection, erythema infectiosum usually appears 2 weeks after the initial infection (16, 41). This is exactly the time from the patient’s first day of admission to the erythema infectiosum appearing on the seven nurses. Blood samples collected from these seven nurses tested positively for DNA, IgM, and IgG of B19, and the highest B19 DNA copy number was 1.0 × 10^3^/ml sera. The virus did not transmit to other people and was self-limited within 2 weeks. This phenomenon further proves that the proportion of typical erythema infectiosum in healthy patients is significantly higher, and the viral load and infectivity are lower (16).

## Conclusion

Once a patient with AH combined with HA is infected with B19, he/she can develop disseminated infection with high viral load, and this can even cause death due to multiple organ failure and can also cause nosocomial transmission of B19 infection. Once fever of unknown origin and aggravation of anemia develop in patients with liver disease and anemia, especially those with a history of blood transfusion, B19 infection should be considered. When collective medical staff have unexplained fever, rash, or arthropathy, B19 may be the culprit.

## Data Availability Statement

The datasets presented in this study can be found in online repositories. The names of the repository/repositories and accession number(s) can be found in the article/**Supplementary Material**.

## Ethics Statement

All clinical data in this case report were either provided by the patient or collected by our team members from the patient with his consent. There was no additional invasive test performed or experimental drugs used incautiously on the patient. Written informed consent was obtained from the patient for participation in the study and the publication of this report in accordance with the Declaration of Helsinki. The case report is exempt from institutional review board approval.

## Author Contributions

JL is the patient’s attending physician, responsible for the management of the patient's entire disease process in the MICU, the conception and writing of this article. JZ is patient’s resident, and is also responsible for the collation of the patient's image data and the editing of the figures in this article.WL is a doctor in the blood laboratory who was responsible for the reading and reporting of the blood and bone marrow smears of this patient. SW and LZ were the nurses in charge of the patient. In addition to clinical care of patient, they were also responsible for recording patient’ clinical data, collecting experimental data related specimens (including blood, urine, sputum, gastric juice, and blister liquid) and providing pictures of patients’ rashes. YS, JB, JH and YW were doctors involved in the diagnosis, treatment, clinical and experimental data interpretation of the patient, and provided suggestions for the conception of the article. LL was responsible for the revision and editing of this article and the PCR detection of some specimens. BW is the PI of the team and the director of the MICU. All authors contributed to the article and approved the submitted version.

## Funding

This work was supported by grants from the Third Affiliated Hospital of Sun Yat-sen University for the Cultivate Special Funding Projects of 2020 National Natural Science Foundation of China (2020GZRPYQN26).

## Conflict of Interest

The authors declare that the research was conducted in the absence of any commercial or financial relationships that could be construed as a potential conflict of interest.

## Publisher’s Note

All claims expressed in this article are solely those of the authors and do not necessarily represent those of their affiliated organizations, or those of the publisher, the editors and the reviewers. Any product that may be evaluated in this article, or claim that may be made by its manufacturer, is not guaranteed or endorsed by the publisher.

## Supplementary Material

Supplementary Table 1 Part of the results of the patient’s blood routine, biochemical, infection and inflammation related tests during the course of the disease, as well as the daily urine output and blood transfusion volume. WBC, white blood cell. RBC, red blood cell. Hb, hemoglobin. MCV, mean corpuscular volume. PLT, Platelet. Ret, reticulocytes. AST, aspartate aminotransferase. ALT, alanine aminotransferase. TBIL, total bilirubin. DBIL, direct bilirubin. IBIL, indirect bilirubin. Cr, creatinine. PTA, prothrombin activity. INR, international normalized ratio. FIB, fibrinogen. CRP, C-reactive protein. PCT, procalcitonin. IL-6, interleukin-6.
